# Acute Pancreatitis Associated With Ketogenic Diet: A Case Report

**DOI:** 10.7759/cureus.57547

**Published:** 2024-04-03

**Authors:** Ibrahim Shanti, Malik Samardali, Leena Alhusari

**Affiliations:** 1 Internal Medicine, Marshall University Joan C. Edwards School of Medicine, Huntington, USA

**Keywords:** elevated serum amylase and lipase, abdominal pain, dietary influence, acute pancreatitis, ketogenic diet

## Abstract

This case study explores the relationship between acute pancreatitis and the ketogenic diet, a dietary approach characterized by low carbohydrate and high fat intake. The report details the experience of a 47-year-old woman who developed intense abdominal pain and vomiting following her self-prescribed ketogenic diet for weight loss. The patient had a medical history of hypertension, depression, and hypothyroidism. Laboratory findings indicated elevated levels of lipase and amylase, confirming the diagnosis of acute pancreatitis. Imaging procedures, including CT scans, further substantiated the diagnosis. The case underscores the potential association between the ketogenic diet and the onset of acute pancreatitis, emphasizing the necessity for healthcare professionals to consider dietary elements in the assessment and treatment of such cases. Additionally, the discussion explores the mechanisms, causes, and complications of acute pancreatitis, shedding light on the increasing interest in the ketogenic diet for weight management and its potential implications for pancreatic health. The study advocates for heightened awareness among healthcare practitioners concerning the risks linked to low-carbohydrate, high-fat diets, urging careful consideration and supervision for individuals contemplating their adoption.

## Introduction

Acute pancreatitis is a severe inflammatory condition of the pancreas that presents with sudden-onset abdominal pain and can lead to serious complications including death. It is often caused by gallstones or excessive alcohol consumption, but other factors such as certain medications, infections, and metabolic disorders can also contribute [1]. The pancreatic exocrine glands produce digestive enzymes called trypsin and chymotrypsin that digest proteins along with lipase that breaks down fats. The self-destruction of pancreatic cells in acute pancreatitis results in the release of these digestive enzymes into the circulation with lipase and amylase blood levels rising three times above the upper limit of the normal range [2]. There are different classification scales of acute pancreatitis, including Atlanta Classification, Revised Atlanta Classification, and Determinant-Based Classification [3]. These scales categorize acute pancreatitis into mild, moderate, or severe acute pancreatitis depending on the presence or absence of pancreatic necrosis and/or organ failure necrosis [3].

The ketogenic dietary regimen mandates a limitation of carbohydrate intake to 5-10% and a fat composition of 70-75% of total daily carbohydrate intake along with 20-25% of protein intake [4]. This specific distribution of macronutrients prompts an elevation in ketone production, notably serving as a primary energy source for the brain. Nonetheless, persistent apprehensions surround the prolonged consequences of the ketogenic diet, particularly concerning cardiovascular and metabolic well-being attributable to heightened saturated fat consumption [4].

However, this diet has shown promise in managing conditions such as epilepsy and obesity, and its impact on pancreatic health remains a topic of investigation. It has been proposed that the high fat content in the keto diet may exacerbate pancreatic inflammation and cause pancreatic damage. We observed a similar case presented here with a 47-year-old female who developed acute pancreatitis after following a ketogenic diet for weight loss. This case highlights the importance of considering dietary factors in the evaluation and management of acute pancreatitis.

## Case presentation

A female patient, aged 47 years, presented at the emergency department (ED) complaining of severe central abdominal pain extending to her back. She reported a general feeling of unwellness over the preceding days and awoke with intense epigastric pain rated at 10/10 in intensity, accompanied by nausea and vomiting. The vomiting, which occurred more than 10 times, involved yellow content but showed no visible signs of blood. Additionally, the patient described chest pain characterized as heavy, relieved by leaning forward, and located centrally. For the past 24 days, she had been adhering to a self-formulated ketogenic diet with a daily caloric intake of 2,200 calories, initiated for weight loss. This marked her initial exposure to such a dietary regimen, resulting in multiple episodes of diarrhea. The patient denied supplement usage and had no history of scorpion bites or previous instances of severe abdominal pain.

Her medical background encompassed hypertension, depression, and hypothyroidism due to Hashimoto thyroiditis, in addition to a history of cholecystectomy. Upon arrival, her vital signs included a blood pressure of 148/103 mmHg, a heart rate of 92 beats/minute, a respiratory rate of 16 breaths/minute, and an oxygen saturation of 96% on room air, with an absence of fever.

The physical examination disclosed a patient in significant distress, rating her pain at 10/10, without observable jaundice. The cardiorespiratory examination was unremarkable, and her abdominal evaluation revealed non-tympanic sounds with normal bowel function and no skin discoloration. Although tender in the epigastrium, her abdomen was otherwise soft, lacking peritoneal signs, right upper quadrant tenderness, or Murphy’s sign. Laboratory findings are presented in Table 1.

**Table 1:** Relevant laboratory results.

Biochemical markers	Reference range	Results
White blood cells (cells/μL)	4,000–11,000	16.1
Creatinine (mg/dL)	0.6–1.2	1.1
Calcium (mg/dL)	8.5–10.5	10.4
Lipase (U/L)	10–140	19,500
Amylase (U/L)	25–125	3500
Lactic acid (mmol/L)	0.5–2.2	0.8
Total cholesterol (mg/dL)	Less than 200	212
Aspartate aminotransferase (U/L)	10–36	30
Alanine aminotransferase (U/L)	4–36	22
Alkaline phosphatase (U/L)	44–145	85
Total bilirubin (mg/dL)	0.1–1.2	0.3
γ-glutamyl transferase (IU/L)	0–30	10

**Figure 1:**
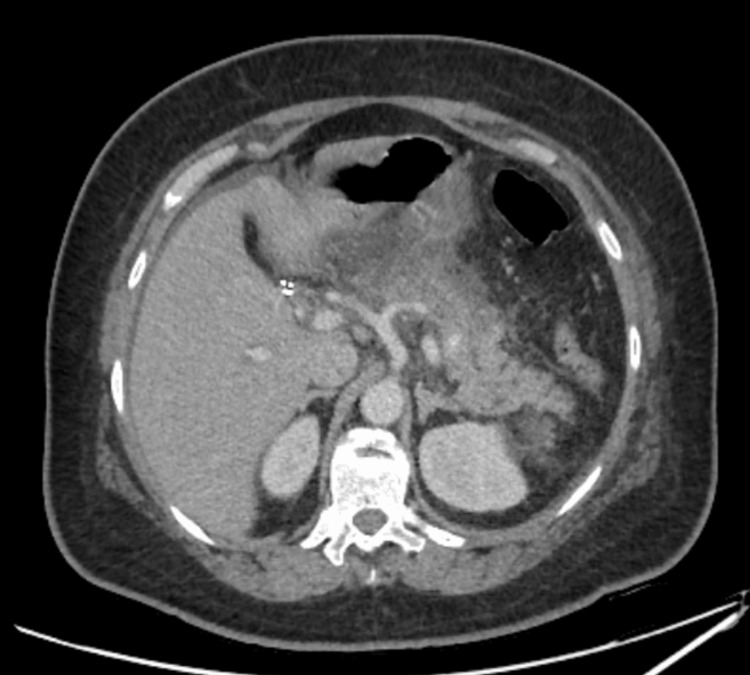
CT scan showing acute pancreatitis.

Urinalysis was negative for urinary tract infection, normal troponin, lipase level was 19,500 U/L, amylase level was 3,500 U/L, and lactic acid was 0.8 mmol/L. A fasting lipid profile showed a total cholesterol of 212 mg/dL, with normal triglycerides. IgG4 negative for suspicion of autoimmune pancreatitis. Chest X-ray revealed no acute cardiopulmonary issues. CT of the abdomen and pelvis with contrast revealed acute pancreatitis with extensive surrounding stranding, while CT angiography showed no pulmonary thromboembolism and mild ground-glass opacity at the lung base, possibly indicating hypersensitive pneumonitis. Abdominal ultrasound showed no discernible ductal dilatation, with a 5 mm common bile duct (Figure 1). Further advanced diagnostic imaging such as magnetic resonance cholangiopancreatography was not indicated in our case.

The patient was diagnosed with acute pancreatitis and received pain and nausea medication, 3 L of fluids, and was kept nothing per mouth for two days. She tolerated a liquid diet and was advanced to a regular diet without complications. She was advised to discontinue the ketogenic diet. At a follow-up visit three weeks later at the gastrointestinal clinic, the patient reported no abdominal pain, nausea, or vomiting and was doing well.

## Discussion

Acute pancreatitis is defined by injury to the acinar cells, which are the exocrine pancreas’ functional units. This leads to the improper release and activation of trypsinogen to trypsin within the acini, initiating the activation of additional digestive enzymes, the kinin system, and the complement cascade. These processes culminate in the self-digestion of the pancreatic tissue [5,6].

In 75-85% of patients, the causes of acute pancreatitis are readily identifiable. In developed countries, the leading causes are obstruction of the common bile duct by stones (38%) and alcohol abuse (36%) [7,8].

The ketogenic diet is a high-fat, low-carbohydrate, adequate protein diet that has been employed as a treatment for medically refractory epilepsy for over 90 years. Due to the increasing prevalence of obesity and diabetes, there is a rising interest in the ketogenic diet as a potential weight loss aid. Few serious complications caused by the diet have been reported. We report acute pancreatitis after starting the keto diet. The case report that marked the first instance of acute pancreatitis associated with the ketogenic diet in a patient with nearly normal triglyceride levels at the time of diagnosis was of a 35-year-old male following a ketogenic diet who presented at the ED experiencing weekly abdominal pain following dietary lapses. His symptoms were diagnosed as acute pancreatitis, and notable factors such as alcohol use, hypertriglyceridemia, pancreatic obstruction, or anatomical abnormalities were absent. Recurring episodes of presumed pancreatitis appeared to be instigated by relatively higher carbohydrate and caloric intake on cheat days, as opposed to the sustained higher fat intake on diet maintenance days. The patient’s triglyceride levels showed significant improvement on the day of his ED presentation compared to the fasting blood work conducted by his primary care physician before commencing the diet. This case underscores the impact of lifestyle and dietary choices on the onset of acute medical conditions, potentially eluding healthcare providers during history-taking. It suggests that the ketogenic diet might decrease the threshold for acute pancreatitis, and an episodic stressor could trigger an acute attack even in the absence of conventional risk factors [9].

The ketogenic diet has been used as an alternative for type 2 diabetes mellitus (T2DM) and has been associated with hypertriglyceridemia and acute pancreatitis. A 19-year-old African American male, maintaining well-controlled T2DM, experienced severe necrotizing pancreatitis induced by hypertriglyceridemia following an unsupervised three-month trial of the diet. The diet’s popularity has heightened awareness of potential adverse effects, emphasizing the importance of caution for patients and healthcare providers. Elevated triglyceride consumption can lead to hypertriglyceridemia, increasing the susceptibility of patients to severe pancreatitis and subsequent harm. Thus, seeking guidance from a registered nutritionist or dietitian is imperative for individuals contemplating the adoption of the ketogenic diet [10].

Ketone bodies have been associated with acute pancreatitis within the context of diabetic ketoacidosis (DKA). DKA is linked with a non-specific elevation in serum amylase levels, and prior autopsy investigations have raised concerns about pancreatic necrosis in DKA patients. A prospective analysis was conducted of 100 consecutive DKA episodes at a New York City university hospital. Patients exhibiting abdominal pain or elevated serum levels underwent a CT scan for diagnosis, and confirmation of acute pancreatitis required the observation of pancreatic enlargement or necrosis on contrast-enhanced CT scans. Among the participants, 11% (11 patients) were diagnosed with acute pancreatitis, attributed to hypertriglyceridemia in four, alcohol in two, drug-induced causes in one, and idiopathic factors in four patients. Lipase elevation occurred in 29%, and amylase elevation in 21% of all DKA patients. The study’s findings suggest that DKA may obscure concurrent AP, with an incidence of at least 10-15%. Furthermore, the severity of acute pancreatitis in DKA is more likely associated with episodes marked by pronounced acidosis and hyperglycemia [11].

With the increasing popularity of low-carbohydrate, high-fat diets, healthcare providers must maintain heightened awareness of potential complications manifesting in emergency settings. This case underscores the profound influence of lifestyle and dietary decisions on acute medical conditions, aspects that healthcare providers might inadvertently disregard in patient assessments [4]. Notably, despite the absence of conventional risk factors or triggers for acute pancreatitis in this instance, we propose that the ketogenic diet may have contributed to the onset of this patient’s condition.

## Conclusions

This case details a 47-year-old woman developing acute pancreatitis on a self-prescribed ketogenic weight-loss diet. While the ketogenic diet proves effective for epilepsy and obesity, its impact on pancreatic health warrants investigation. The case contributes to mounting evidence suggesting a possible link between the ketogenic diet and acute pancreatitis, urging healthcare providers to consider diet in managing such cases. With identifiable causes such as gallstones or alcohol, acute pancreatitis is now linked to the rising popularity of ketogenic diets, emphasizing the need for heightened awareness. As the diet gains traction for health goals, caution is crucial for patients and healthcare providers, prompting further research into potential connections with acute pancreatitis.
